# Measurement for the Thickness of Water Droplets/Film on a Curved Surface with Digital Image Projection (DIP) Technique

**DOI:** 10.3390/s20082409

**Published:** 2020-04-23

**Authors:** Lingwei Zeng, Hanfeng Wang, Ying Li, Xuhui He

**Affiliations:** 1 School of Civil Engineering, Central South University, Changsha, Hunan 410075, China; 174812223@csu.edu.cn (L.Z.); wanghf@csu.edu.cn (H.W.); xuhuihe@csu.edu.cn (X.H.); 2 National Engineering Laboratory for High-speed Railway Construction, Central South University, Changsha, Hunan 410075, China; 3 Department of Mechanical Engineering, The Hong Kong Polytechnic University, Hung Hom, Kowloon, HongKong Special Administrative Region 999077, China

**Keywords:** water droplets/film, curved surface, digital image projection DIP, image processing technique

## Abstract

Digital image projection (DIP) with traditional vertical calibration cannot be used for measuring the water droplets/film on a curved surface, because significant systematic error will be introduced. An improved DIP technique with normal calibration is proposed in the present paper, including the principles, operation procedures and analysis of systematic errors, which was successfully applied to measuring the water droplets/film on a curved surface. By comparing the results of laser profiler, traditional DIP, improved DIP and theoretical analysis, advantages of the present improved DIP technique are highlighted.

## 1. Introduction

The properties of water droplets/film on a surface are of great significance in the aviation engineering, civil engineering, thermal engineering, etc. Icing is widely considered as one of the most serious weather hazards to aircraft. When an aircraft flies through clouds or fog containing super-cooled small water droplets, icing will occur after those droplets impact on the airfoil (Mason [1]). Under incompletely freezing conditions, only a fraction of the water freezes in the impact area. Other water runs back, driven by the boundary flow over the surface. This phenomenon results in the change of water distribution on the airfoil, forming glaze ice outside the impact area (Politovich [2], Hu and Huang [3] and Hu and Jin [4]). The shape of glaze ice is complicated and hard to predict accurately, which may significantly deteriorate the aerodynamic performance of airfoil.

Stay cables of bridge are prone to large amplitude vibrations especially when rain and wind occur simultaneously, which is called rain–wind-induced vibration (RWIV). The upper rivulet has significant influences on the RWIV of the cable since its circumferential movement shifts the flow around the cable from subcritical to supercritical regions alternately (Seidel and Dinkler [5]). Quantitative information of the water rivulets, including thickness, width and position, is essential to reveal the mechanisms of RWIV (Flamand [6] and Du et al. [7]).

The measurement of rivulets is also important in the thermal engineering. When a rivulet flows on a solid surface, it shares an interface with the surrounding gas. The rivulet is crucial for investigating heat exchange and gas absorption process (Rekstad et al. [8]). Moreover, it is also indispensable to obtain the precise information of the liquid film flow on nuclear fuel rods when analyzing the critical heat flux under postulated loss-of-coolant accidents in boiling water reactors (Hoshiide et al. [9] and Yano et al. [10]).

Recently, Hu et al. [11] and Zhang et al. [12] proposed a digital image projection (DIP) technique, which was successfully used to measure the instantaneous pattern of water rivulets and film on a NACA0012 aerofoil with the chord length of 101 mm.

**Figure 1:**
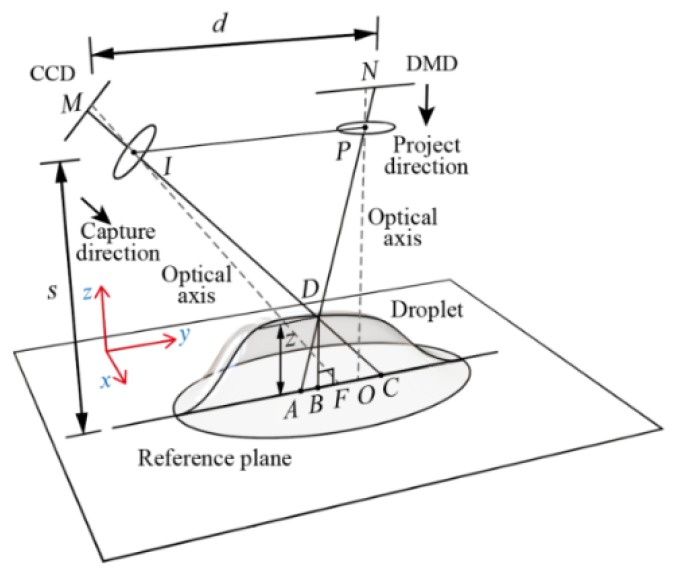
Schematic diagram of the digital image projection (DIP) technique (Zhang et al. [12]).

The principle of the DIP technique is briefly introduced for readers here. As shown in Figure 1, when there is no droplet on the plane, a projected image from the projector (point N) falls on point A. If an opaque droplet appears on the plane, the light will fall on its surface at point D. However, from the view of the camera (point M), the image moves to point C. The distance between A and C, i.e., $\bar{\mathrm{AC}}$, can be determined based on cross-correlation between the two projected images taken with and without the droplet.

Assume that the horizontal distance between the projector and the camera is *d*, the reference plane is parallel to them with a distance *s*. From the relationship between ΔMND and ΔACD, the following relation applies:

(1) $$\frac{d}{\bar{\mathrm{AC}}\;}=\frac{s-\bar{\mathrm{BD}}}{\bar{\mathrm{BD}}}\;\approx \;\frac{s}{\bar{\mathrm{BD}}}$$

(the distance *s* is usually much larger than $\bar{\mathrm{BD}}$).

According to this relation, the thickness of the droplet, *z* (*x*, *y*), can be obtained:

(2) $$z\left(x,\;y\right)=\bar{\mathrm{BD}}\;\approx \;\frac{s}{d}\bar{\mathrm{AC}}{=k}_{1}\bar{\mathrm{AC}}$$

The conversion coefficient *k*_1_, i.e., the displacement-to-height conversion constant, can be determined through a so-called “vertical calibration procedure” for a given DIP system. By utilizing vertical calibration procedure, the coefficient *k*_1_ in Equation (2) can be obtained. 

However, the traditional DIP method assumes the area, on which the water rivulet is measured, is flat or of exceedingly small curvature (e.g., an aerofoil). Thus, the calibration is only conducted in the direction perpendicular to the plane (Hu et al. [11] and Zhang et al. [12]). Consequently, the traditional DIP technique cannot be applied directly to a surface with large curvature. Otherwise, significant systematic error will be introduced. The present paper proposes a new calibration method to overcome this issue, which allows the DIP technique to be applied to curved surface.

## 2. Technical Principles of the Improved DIP Method

### 2.1. Methodology

**Figure 2:**
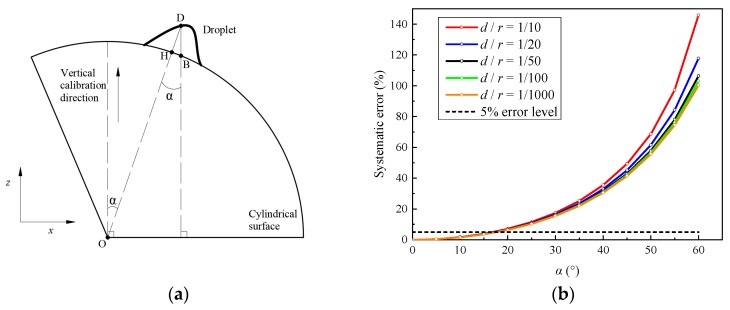
Schematic diagram of (**a**) a droplet on the cylindrical surface and (**b**) dependence of the systematic error on *α* for different *d*/*r*.

For the measurement on a curved surface (as shown in Figure 2a), if the preceding vertical calibration is still adopted, the measured thickness $\bar{\mathrm{DB}}$ will be in the vertical direction. However, the real thickness of the droplet is $\bar{\mathrm{DH}}$, which is normal to the surface. Obviously, as illustrated in Figure 2b, the systematic bias between $\bar{\mathrm{DB}}$ and $\bar{\mathrm{DH}}$ depends on the included angle α between them. With the increase of *α*, the systematic bias raises quickly. Moreover, the ratio of the real thickness *d* ($\bar{\mathrm{DH}}$) and the local radius of the curved surface *r* also contributes to the systematic bias. This effect will be magnified when *α* becomes larger. That is, the traditional DIP overestimates the thickness of droplets on a curved surface. If the accepted error level is 5%, the traditional DIP technique must be improved to eliminate this systematic error when *α* is larger than 17°, even when *d*/*r* approaches zero.

**Figure 3:**
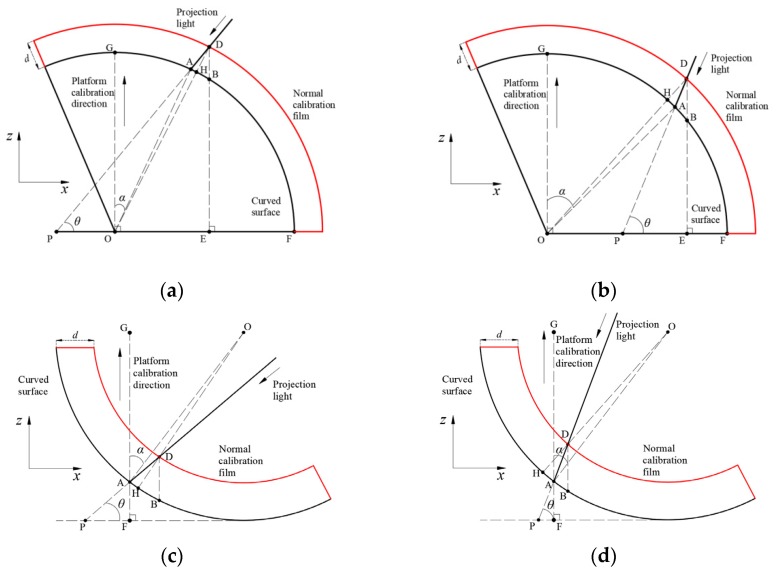
Typical geometric configurations of the DIP measurement on curved surface: (**a**) 90°−*α* ≥ *θ* (convex surface); (**b**) 90°−*α* ≤ *θ* (convex surface); (**c**) 90°−*α* ≥ *θ* (concave surface); (**d**) 90°−α ≤ θ (concave surface)

Consequently, a correction coefficient $k_{2}$ (= $\frac{\bar{\mathrm{DH}}}{\bar{\mathrm{DB}}}$) is necessary to eliminate the above-mentioned bias for applying the DIP technique on a curved surface. Figure 3a shows a typical geometric configuration of the DIP measurement on a curved surface. The projected light falls on an arbitrary curved surface at point A with an angle *θ*. The angle between radius $\bar{\mathrm{OA}}$ and the vertical direction, $\bar{\mathrm{OG}}$, is *α*. It should be mentioned that *α* can be negative when the projected light falls on the left side of $\bar{\mathrm{OG}}$.

If the original curved surface expands to its new position with a normal displacement of *d* ($\bar{\mathrm{DH}})$, as shown in Figure 3a. Assume that the radius of the curved surface *r* is much larger than *d*. Under the condition of 90°−*α* ≥ *θ*, the thickness $\bar{\mathrm{BD}}$, acquired by the previous vertical calibration procedure, can be calculated by Equations (3)–(5):

(3) $$\bar{\mathrm{BD}}=\left(r+d\right)\cdot \cos∠\mathrm{ODE}\;-\bar{\;BE}$$

(4) $$\bar{BE\;}=\sqrt{r^{2}-{\left[\left(r+d\right)\cdot \sin∠\mathrm{ODE}\right]}^{2}}$$

(5) ∠ODE=∠ADE−∠ADH=90°−θ−∠ADH

According to the law of sines:

(6) $$\frac{\bar{\mathrm{OA}}}{\sin(∠\mathrm{ADH})}=\frac{\bar{\mathrm{DH}\;}+\bar{\mathrm{HO}}}{\sin(∠\mathrm{OAD})}$$

Because ∠OAP+∠OAD=180° and ∠OAP=90°−α−θ, thus:

(7) sin(∠OAD)=sin(∠OAP)=sin(90°−α−θ)

Hence, Equation (6) can be simplified according to Equation (7) as follows:

(8) $$∠{\mathrm{ADH}=\sin}^{-1}[\frac{r\cdot \sin\left(90{}^{\circ}-\alpha -\theta \right)}{r+d}]$$

Based on the discussion above, in a certain interrogation window on the surface (i.e., *r*, *α* and *θ* are constant), the thickness, $\bar{\mathrm{BD}}$, is only determined by the normal displacement, *d*. Meanwhile, the correction coefficient $k_{2}$ (=$\;\frac{d}{\bar{\mathrm{BD}}}\;$) can be also determined.

Figure 3b–d present the other three possible configurations for the application of the DIP measurement on a curved surface. Based on similar analysis procedure, *k*_2_ for these configurations can all be determined.

**Figure 4:**
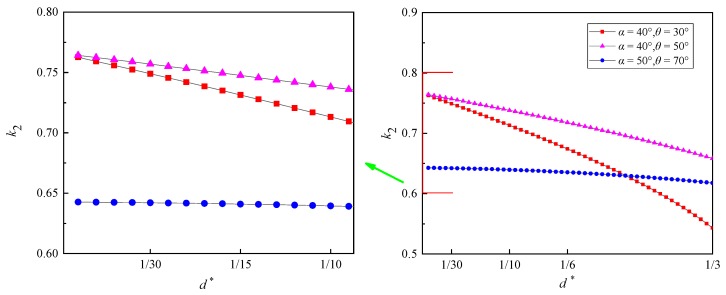
Relationship between d* and the corrected coefficient *k*_2_.

Figure 4 illustrates the relationship between d^*^ (normalized with the local radius of the curved surface *r*) and *k*_2_, calculated from Equations (3)–(8). *k*_2_ decreases gradually with the increasing d^*^. Obviously, $k_{2}$ and d^*^ show good linear relationship when d^*^ ranging from 0 to 0.1.

Assume that the linear relation between d^*^ and *k*_2_ is:

(9) $$k_{2}=\frac{d^{*}}{{\bar{\mathrm{BD}}}^{*}}=\frac{d}{\bar{\mathrm{BD}}}{=C}_{1}\cdot d^{*}{+C}_{2}{=C}_{3}\cdot {d+C}_{4}$$

where ${\bar{\mathrm{BD}}}^{*}$ is normalized with the local radius of the curved surface *r*.

According to Equation (2), Equation (9) can be expressed as:

(10) $$\frac{d}{\bar{\mathrm{AC}}}{=k}_{1}\cdot {\;C}_{3}\cdot {d+k}_{1}\cdot C_{4}\;$$

In a certain interrogation window on the surface, *k*_1_ is a constant (Zhang et al. [12]). Hence, Equation (10) indicates that $\frac{d}{\bar{\mathrm{AC}}}$ and *d* are also quasi-linearly correlated. Thus, it is acceptable to get the $\frac{d}{\bar{\mathrm{AC}}}-d$ relation by measuring $\bar{\mathrm{AC}}$ at only two specific *d*.

If we can attach two shells with given thickness to the curved surface, respectively, then the linear relation between $\frac{d}{\bar{\mathrm{AC}}}$ and *d* can be acquired. This calibration is named for “normal calibration” hereafter in the present paper. It can be obtained:

(11) $$d=\frac{{-\;C}_{4}\cdot k_{1}\cdot \bar{\mathrm{AC}}}{{\;C}_{3}\cdot k_{1}\cdot \bar{\mathrm{AC}}\;-\;1}$$

In Equation (11), the distance $\bar{\mathrm{AC}}$ between the two images can be determined based on cross-correlation between the tested images and reference image. The constant coefficients *k*_1_⋅*C*_3_ and *k*_1_⋅*C*_4_ can be calculated by normal calibration procedure. Hence, the normal displacement, *d*, can be also computed by Equation (11). 

### 2.2. Error Analysis

**Figure 5:**
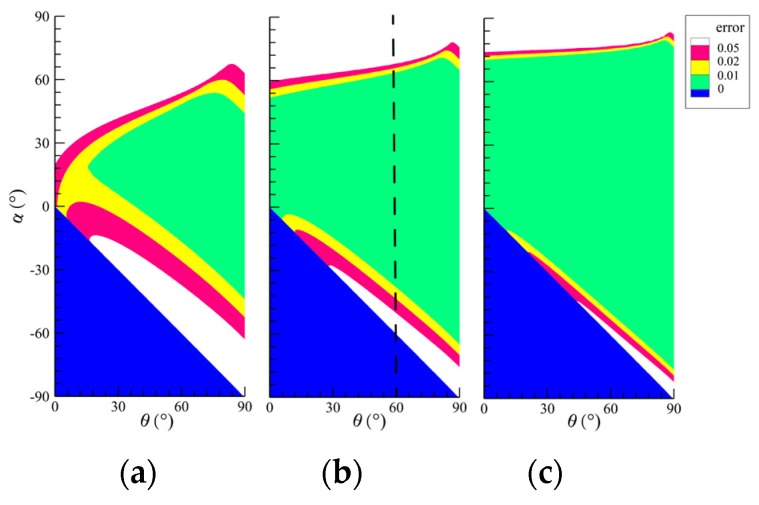
Maximum systematic error with different combinations of θ and α: (**a**) *d*_max_/*r* = 1/3; (**b**) *d*_max_/*r* = 1/10; (**c**) *d*_max_/*r* = 1/33.

The $k_{2}-d^{*}$ in normal calibration is assumed to be perfectly linear. The systematic error caused by this assumption is related to *α*, *θ* and the ratio of *d*_max_ (the maximum normal displacement) and *r*. By optimizing the setup arrangement, e.g., *α* and *θ*, this systematic error can be controlled within an acceptable level. Figure 5 shows the dependence of this systematic error on the two main influencing factors *α* and *θ* for different *r* and *d*_max_. The thickness of two hypothetical shells for normal calibration is set as $\frac{1}{3}d_{\max}$ and $\frac{2}{3}d_{\max}$.

**Figure 6:**
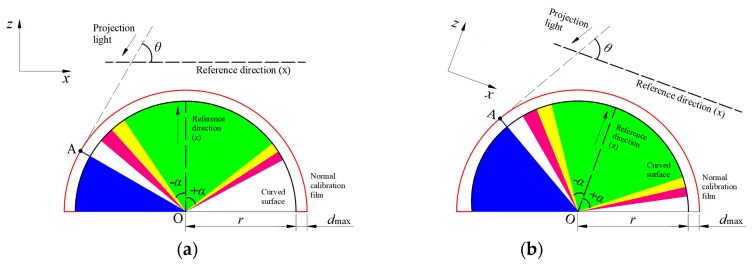
Systematic error under the condition of *d*_max_/*r* = $\frac{1}{10}$, θ = 60° utilizing (**a**) standard reference axis or (**b**) arbitrary reference axis.

For better illustration, Figure 6 displays the systematic error under the condition of *d*_max_/*r* = $\frac{1}{10}$,θ = 60°, marked with the black dashed line in Figure 5b. Note that, as shown in Figure 3, *θ* is determined by the position and the incidence angle of the projector and *α* represents the location of the interrogation window to be tested. As expected, both *α* and *θ* have obvious effects on the systematic error. In Figure 6, the blue region on the left side of OA is out of the field illuminated by the projected light. Based on the information provided by Figure 5 and Figure 6 and Equation (1), the projector should be mounted far away from the reference surface. Meanwhile, in order to obtain a larger green area, the recommended angle θ is about 80°. Moreover, rather than the standard reference axis (vertical direction and horizontal direction) reported by Zhang et al. [12], as shown in Figure 6a, the reference axis can be adjusted arbitrarily, making the tested region within the green area in order to obtain precise results.

Likewise, the ratio of *d*_max_ and *r* also has remarkable influences on the systematic error. According to Figure 5, the systematic error reduces with the decrease of the ratio of *d*_max_ and *r* and vice versa. 

### 2.3. Experimental Setup

**Figure 7:**
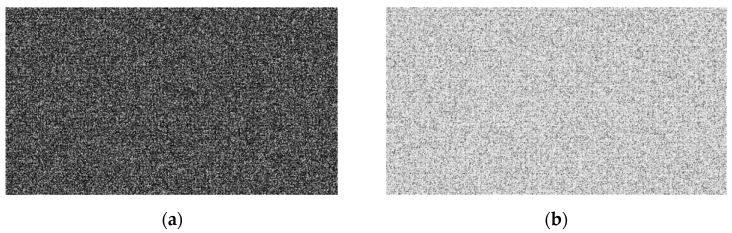
Projected image with random scattered particles: (**a**) low grayscale; (**b**) high grayscale.

A grayscale image consists of particles with random gray values generated by Matlab was used as the projected image, as shown in Figure 7. The gray level of this projected image should be adjusted according to the ambient lighting condition. Because the combined effects of intense ambient lighting condition and high gray level may lead to the improper focusing of the camera. Thus, it is recommended that the experiments are conducted in a dark environment. Meanwhile, the gray level of the projected image should not be too high.

In the present experiment, a digital projector (Robot go 3D, produced by Aiteqi Technology Company Limited, Shenzhen, China; the resolution was 1280 × 800 pixel^2^) was used to cast the random scatter particle image onto a reference surface for the DIP measurement. And a digital single-lens reflex (DSLR) camera (Canon EOS 6D, produced by Canon Company Limited, Tokyo, Japan; the highest resolution was 5472 × 3648 pixel^2^, the valid pixel was 20 million) was applied for capturing images. The measured surface was one-third of a cylindrical surface with the length of 70 mm and radius of 50 mm. It was produced by a rapid 3D prototyping machine using white resin material and carefully polished. Two normal calibration shells, with the thickness of 1 mm and 2 mm, respectively, were prepared.

A high-resolution 2D Laser Profiler (Keyence LJ-G200, produced by Keyence Corporation, Itasca, IL, USA; the length of the linear laser was 62 mm, the accuracy was up to 2 μm) was also used to measure the thickness of the droplet, which provided the validation for the present DIP technique.

The normal calibration procedure can be divided into the following steps:

1. Based on the measurement region, fix the projector and camera according to the result shown in Figure 5 and Figure 6.
2. Project the image onto the tested curved surface. Take a picture of the projected image without water droplet or rivulet, which will be used as “original image”.
3. Attach the two thin shells with given thicknesses to the measured surface, respectively. Take pictures of the projected image on each of the two shells. These two pictures will be used as “normal calibration images”.
4. Remove the thin shells. Make droplets or rivulets attached on the surface. Then, the projected image will be distorted with the appearance of these water droplets or rivulets. Capture the distorted images, which contain the geometric information of the water droplets or rivulets.
5. Calculate the constant coefficients *k*_1_⋅*C*_3_ and *k*_1_⋅*C*_4_ with cross-correlation between the “normal calibration images” and “original image”.
6. Calculate the thickness of water droplets or rivulets with cross-correlation between the distorted images and “original image”

Noted that, in order to enhance the contrast of the projected image on the free surface of the water droplet, a very small amount of titanium pigment should be added into the water. 

### 2.4. Interrogation Window Size

The interrogation window (IW) size does influence the measurement results. For the present experiment, the resolution of the camera was 5472 × 3648 pixel^2^ (which was not completely used for cross-correlation, only the part containing the droplet was adopted for the measurement), each pixel indicates 0.05 mm. The cross-correlation algorithm provides one result for each IW according to the image displacement within it. It was similar to a spatial filtering or average for each IW. Thus, a larger IW reduces the spatial resolution of the measurement. However, if the IW is set to small, the cross-correlation algorithm may not provide a reliable result, because the image within each IW is too small to provide enough information for cross-correlation. The smallest applicable IW largely depends on the quality of the image captured.

**Figure 8:**
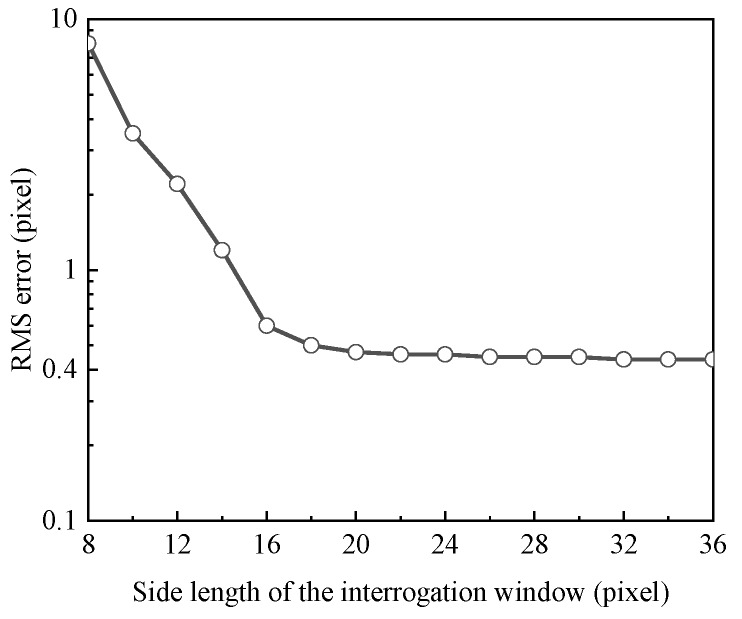
Root Mean Square (RMS) error as function of the interrogation window size.

For the present experiment, the effects of IW on the results were tested during calibration process. Since the thickness of the calibration shell was given and uniform, the cross-correlation was conducted for different IWs and their corresponding RMS values were calculated, as shown in Figure 8. As expected, the Root Mean Square (RMS) value reduces quickly with increasing the size of IW and becomes stable for the IW larger than 18 × 18 pixel^2^ (0.9 × 0.9 mm^2^). Consequently, the IW of 18 × 18 pixel^2^ was utilized in the experiment.

## 3. Applications

### 3.1. Measurement for a Curved Uniform Shell

**Figure 9:**
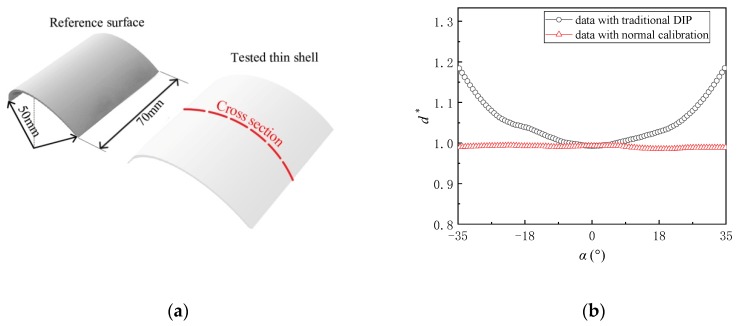
Measurement for a curved shell: (**a**) experimental models; (**b**) results along the central cross section.

The present DIP technique was utilized to measure a curved uniform thin shell with a given thickness of 1.5 mm to test its applicability. The reference surface was one-third of a cylindrical surface with the length of 70 mm and radius of 50 mm (the same with that introduced in Section 2.3), as shown in Figure 9a. The tested thin curved shell was attached firmly on the reference surface. Figure 9b shows the results along the central cross-section (marked with a red dashed line in Figure 8a obtained by the DIP technique with the traditional vertical calibration and the present normal calibration, respectively. The result was normalized with the given thickness of the tested shell (1.5 mm). Obviously, the thickness obtained by the traditional DIP method increases with the absolute value of *α*, while the present normal calibration technique provides more accurate results within *α* from −35° to 35°.

### 3.2. Measurement for the Water Droplet on a Flat Surface

**Figure 10:**
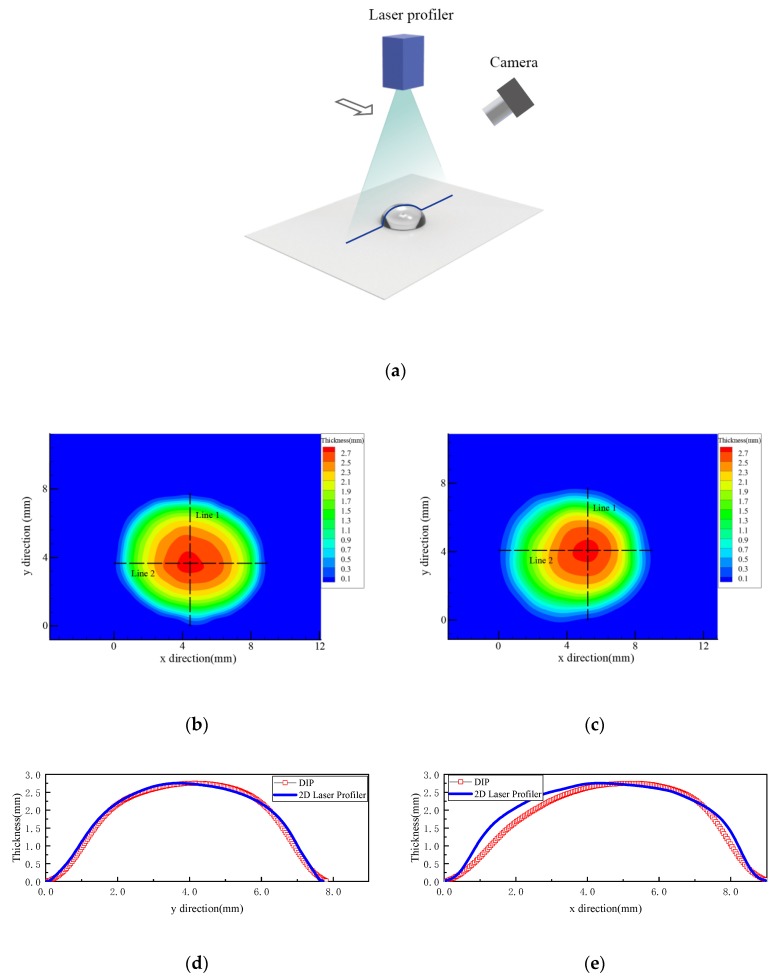
Measuring results of a droplet on the flat plane: (**a**) droplet on a flat surface; (**b**) result of 2D laser profiler; (**c**) result of DIP; (**d**) thickness along Line 1; (**e**) thickness along Line 2.

A high-resolution 2D Laser Profiler (Keyence LJ-G200, produced by Keyence Corporation, Itasca, IL, USA;) was used to measure the thickness of a droplet to validate the present DIP method. The measurements were conducted on a flat and a cylindrical surface, respectively. The Laser Profiler was mounted on a 2D traverse system with an accuracy of 0.02 mm, which was used to move the Laser Profiler to scan the droplet, as shown in Figure 10a. Figure 10b,c present the measurement results of the droplet on a flat surface using the Laser Profiler and DIP technique, respectively. Figure 10d,e qualitatively compare the thickness along the two typical lines (Line 1 and Line 2 in Figure 10b,c, which cross the apex of the droplet. The measurement results using the above mentioned two techniques accord quite well with each other, except for the slight distortion of the DIP result along Line 2. The distortion in the horizontal direction was caused by the slant arrangement of the camera in that direction, as shown in Figure 10a. This distortion of DIP technique can be neglected when applying to large area measurement (Hu et al. [11] and Zhang et al. [12]). Apparently, it can be confirmed that the DIP technique is applicable for measuring the droplet/rivulet on a flat surface. 

### 3.3. Measurement for the Water Droplet on a Curved Surface

**Figure 11:**
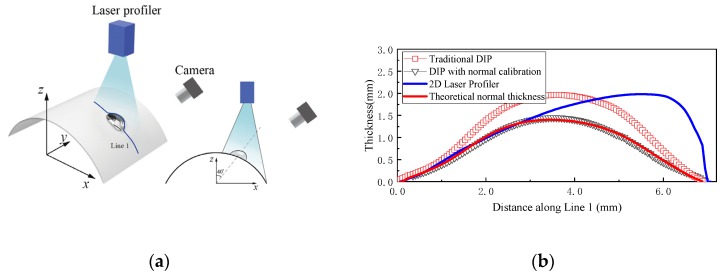
Measurement results of a droplet on the cylindrical surface: (**a**) sketch of the experimental setup; (**b**) thickness along Line 1.

Figure 11 compares the measurement results of a droplet on the cylindrical surface (identical to that introduced in Section 2.3) for the three different approaches, respectively. The droplet was located at the position with an included angle α of about 40°, as shown in Figure 11a. The measurement results of the droplet along Line 1 in Figure 11a are compared in Figure 11b for the three approaches. Obviously, the measurement results acquired by different methods have different features, which are accordant with their measurement principle and limitation, respectively. Laser profiling measurement technique gives the distance along the direction of laser axis, similar to the calibration direction of traditional DIP technique. Unlike a flat surface, the normal direction is different at different points on a curved surface. Therefore, both laser profiler and traditional DIP can only measure the thickness along vertical direction. That was the reason for nearly the same maximum thickness (1.97 mm for Laser Profiler and 1.95 mm for traditional DIP) measured by these two methods. This observation suggests again that the traditional DIP and Laser Profiler will overestimate the real thickness of the droplets/film if they are on a curved surface. Meanwhile, because of slant arrangement of the camera (as illustrated in Figure 11a), it was not unexpected that the apex lies in the middle part of the droplet from the view of the camera.

For improved DIP with normal calibration, the measured maximum thickness was only about 1.48 mm—far smaller than that measured by traditional DIP method. In order to verify the accuracy of the normal calibration according to the vertical thickness acquired by traditional DIP and correction coefficients *k*_2_ calculated by Equations (3–8), the theoretical normal thickness was obtained, shown in Figure 11b, marked with red line. The maximum error of the thickness between the present improved DIP and theoretical normal thickness was about 0.06 mm. Therefore, the proposed DIP with normal calibration technique presents its superiority and accuracy when applied for measuring the droplets/film on a curved surface. 

## 4. Conclusions

In this study, an improved digital image projection (DIP) technique with normal calibration was developed based on the traditional DIP techniques (Hu et al. [11] and Zhang et al. [12]). This improved DIP technique may be used to measure the thickness of droplets/film on curved surfaces. If the accepted error level is 5%, the traditional DIP technique must be improved to eliminate systematic error when α is larger than 17°, even when *d*/*r* approaches zero. Based on theoretical analyses and experimental investigations, it can be concluded that normal calibration can be realized by attaching two thin shells with given thickness to the tested curved surface, respectively. The improved DIP with normal calibration corrects systematic error caused by applying the traditional DIP to the curved surface, which can successfully provide accurate thickness in normal direction for tested objects, e.g., droplets/film.
